# Defining benefit: Clinically and biologically meaningful outcomes in the next‐generation Alzheimer's disease clinical care pathway

**DOI:** 10.1002/alz.14425

**Published:** 2024-12-19

**Authors:** Aya Elhage, Sharon Cohen, Jeffrey Cummings, Wiesje M. van der Flier, Paul Aisen, Min Cho, Joanne Bell, Harald Hampel

**Affiliations:** ^1^ Eisai Inc. Nutley New Jersey USA; ^2^ Toronto Memory Program North York ON Canada; ^3^ University of Nevada Las Vegas Las Vegas Nevada USA; ^4^ Alzheimer Center Amsterdam, Neurology, Vrije Universiteit Amsterdam, Amsterdam UMC Amsterdam The Netherlands; ^5^ Amsterdam Neuroscience, Neurodegeneration Amsterdam The Netherlands; ^6^ Epidemiology and Data Science, Vrije Universiteit Amsterdam, Amsterdam UMC Amsterdam The Netherlands; ^7^ Alzheimer's Therapeutic Research Institute, University of Southern California San Diego California USA

**Keywords:** activities of daily living, Alzheimer's disease, amyloid, biomarkers, clinical meaningfulness, clinical trial, cognition, cognitive impairment, dementia, disease‐modifying therapies, MCI, mild cognitive impairment, multi‐modal assessment, quality of life

## Abstract

To understand the potential benefits of emerging Alzheimer's disease (AD) therapies within and beyond clinical trial settings, there is a need to advance current outcome measurements into meaningful information relevant to all stakeholders. The relationship between the impact on disease biology and clinically measurable outcomes in cognition, function, and behavior must be considered when defining the meaningful benefit of early AD therapies. In this review, we discuss: (1) the lack of consideration for biomarkers in the current concept of meaningfulness in AD; (2) the lack of gold standards for determining minimal biologically and clinically important differences (MBCIDs) in AD trials; (3) how the treatment benefits of disease‐modifying treatments are cumulative and increase over time; and (4) the different concepts of meaningfulness among key stakeholders. This review utilizes the future clinical biological framework of AD and aims to further integrate and expand the parameters of meaningful benefits toward a precision medicine framework.

**Highlights:**

- Definition of meaningful benefit from Alzheimer's disease (AD) treatment varies across disease stage and stakeholder perspectives.
- Observable and meaningful outcomes must consider the clinical‐biological nature of AD.
- Statistically significant effects or outcomes do not always equate to clinically meaningfulness.
- Assessment tools must reflect stage‐specific subtle changes following treatment.
- Real‐world evidence will support consensus, definition, and interpretation of clinical meaningfulness.

## 1 THE UNDERLYING BIOLOGY OF ALZHEIMER'S DISEASE AND THE NONLINEARITY OF DISEASE PROGRESSION

Alzheimer's disease (AD) is a complex, chronic, non‐linear progressive brain proteinopathy that develops from a pathological perspective to a primary neurodegenerative disease, affecting cognition, function, and behavior at later phenomenological stages.^1^ Manifestations of clinical symptoms—and ultimately of the dementia syndrome in AD—are preceded by specific pathophysiological changes in distinct brain regions. These manifest as the accumulation, aggregation, and deposition of soluble amyloid species to form plaques, as well as the accumulation of phosphorylated tau (p‐tau) within neurons forming neurofibrillary tangles.^1^ Historically, late‐stage (e.g., dementia‐stage) clinical manifestations of the disease have guided AD detection, diagnosis, and management, with overt and clinically observable signs and symptoms defining the progression of the disease. With the advent of biological elucidation of AD pathophysiology, the AD framework has been expanded to preclinical asymptomatic stages and early detection. As such, diagnoses have been reconceptualized and defined by core pathophysiological biomarkers that reflect the underlying biology of the disease.

In this context, the definition and parameters of how disease may be modified—and to what extent modifications are impactful versus measurable—are crucial for researchers and clinicians in understanding the development of new therapies that target underlying pathophysiological changes. Disease‐modifying therapies (DMTs) are therapeutic agents that may have an enduring impact on the biological course of disease, and thus may result in beneficial clinical outcomes (i.e., by preserving function, slowing disease progression, and delaying time to disease milestones).^2^ Historically, trial designs have utilized “DMT” terminology to differentiate from previously approved treatments that were conceptualized as “symptomatic” (e.g., cholinesterase inhibitors). Given the information available at the time, such terminology provided a pragmatic model for research and development purposes. In this theoretical context, it should be considered that effective biologically active treatments of complex diseases at specific time points in specific individuals will be best characterized by dimensional scales and will differentiate from dichotomous definitions of effective versus non‐effective, or symptomatic versus disease modifying. The methodological research question remains how dimensionalities can be best operationalized for clinical trials and outcomes.

At present, however, there is a need to reevaluate these concepts so that they are centered more around the complex and comprehensive understanding of biomarker‐guided, targeted therapies that have an impact on both the clinical and biological course of the disease. In the context of AD, there is a lack of precise criteria to distinguish one class of therapeutics from another. Although the newer generation of investigational compounds is often considered to be biomarker‐guided, neuroprotective interventions targeting underlying disease mechanisms,^2^, ^3^ the use of terms such as “biomarker‐guided therapies” or “molecular pathway‐targeting therapies” may better convey the richness and subtlety of emerging classes of drugs in AD. In the following sections, we use the term “DMTs” to address current therapeutics as referenced and discussed in the existing literature, whereas “biomarker‐guided therapies” will be used when alluding to future therapeutics and associated concepts. Ultimately, this terminology must be further differentiated to better describe the evolution of treatment options and combinations for diverse biological subgroups of patients in future clinical trials and the achievement of precision medicine in neurology and psychiatry.^4^

It is critical that clinicians understand the potential benefits of new therapies and their effects on AD‐related biological changes, and better recognize treatment efficacy beyond measures used with previously approved AD treatments. Moreover, treatment decisions should be better informed by available biomarkers and diagnostic assessments that match the evolving understanding of AD. Implementing clear definitions and collecting necessary evidence will facilitate proper care of individuals treated with DMTs for AD and enhance the understanding of the precise meaningfulness and treatment effects such agents exert.

The current concept of clinical meaningfulness—which can be defined as descriptive and observable effects of treatment on aspects of cognition, function, and/or behavior^5^—is limited and fails to take into account underlying biology, nosology, and patient centricity. In addition, finding consensus as to what constitutes treatment benefit for individuals with AD is challenging due to the complex and varied nature of the disease, different perspectives of important stakeholders, and the influence of measures and analyses used to explore meaningfulness. For instance, sporadic AD follows a nonlinear and dynamic progression pattern across a complex disease continuum. In AD, the clinical presentation of signs, symptoms, and diverse pathophysiological alterations affect different biological systems at different times. Therefore, an integrative, comprehensive, and multidimensional approach, taking into account spatial and temporal (ideally staging) information, is needed to define what constitutes meaningful biological and clinical benefits associated with biomarker‐guided targeted therapies for AD.^6^

Herein we explore a comprehensive and more holistic concept of clinical and biological meaningfulness to encompass biomarkers. This discussion contrasts from the traditional framework, which is based largely on clinical descriptive phenomenology. The aims of this article are to (1) articulate the need to redefine clinical meaningfulness to account for both clinical and biological information; (2) highlight that there are no gold standards for determining minimal biologically and clinically important differences (MBCIDs) in AD trials (and that existing approaches have limitations); (3) demonstrate that the treatment benefits of DMTs are cumulative and expected to increase over time; and (4) emphasize that the concept of meaningful benefits differs among key stakeholders (e.g., patients, physicians, care partners, and payors/regulators).

## 2 STAGING ACROSS THE ALZHEIMER'S DISEASE CONTINUUM

The National Institute on Aging and Alzheimer's Association (NIA‐AA),^7^ U.S. Food and Drug Administration (FDA),^8^ and an international working group (IWG)^9^ have each proposed various AD staging systems. The latest 2024 update^10^ of the NIA‐AA 2018 research framework^7^ further defines the disease by combining clinical and biological staging to separate syndrome from etiology. All of these staging schemes categorize the disease based on a combination of the presence or absence of biomarker changes.^7^, ^8^, ^9^, ^11^, ^12^ Some approaches are based exclusively on biomarkers, whereas others include clinical manifestations plus biomarkers. For example, preclinical AD is defined as an individual who is cognitively normal but with biomarker evidence of AD pathology, whereas mild cognitive impairment due to AD (MCI‐AD) refers to an individual with symptomatic cognitive changes, maintenance of functional independence, and biomarker evidence of AD. Individuals with preclinical or MCI‐AD with similar phenotypes may have varying combinations of biomarkers of amyloid, tau, and neurodegeneration. Dementia of the AD type, meanwhile, applies to individuals with cognitive deficits, loss of functional independence, and biomarker(s) indicative of AD pathology.

The presentation and progression patterns of AD are variable due to a multifactorial integration of environment, aging, senescence, genetic factors, cognitive reserve and resilience, and the gradual failure of adaptive and compensatory biological mechanisms.^13^ The time spent at each stage along the nonlinear AD continuum is also inherently heterogeneous.^14^ Based on estimations using patient‐level data from six longitudinal cohort studies, the estimated stage‐specific duration has substantial ranges: 7.6–13 years in preclinical AD, 4.0–4.6 years in MCI‐AD, 2.1–5.0 years in mild AD dementia, and 1.7–6.5 years in moderate to severe AD dementia.^14^

**RESEARCH IN CONTEXT**

1. **Systematic review**: In the context of clinical observations and biological impact, the authors review the literature and describe meaningful benefits of Alzheimer's disease (AD) therapeutics as a concept of minimal biologically and clinically important differences.
2. **Interpretation**: We note important theoretical assumptions for AD disease‐modifying therapies where the extent of biological/clinical intactness to be a function of time — of treatment onset and treatment duration— both of which must be reflected in the non‐linear progression of disease with time‐dependent cumulative changes in underlying pathophysiology and complexity.
3. **Future directions**: We propose a framework for future consideration of evaluating clinical meaningfulness of AD therapeutics.

In individuals with preclinical AD, clinical symptoms are usually absent, too subtle to be recognized by community clinicians, or appear as subjective cognitive decline (SCD) exclusively perceived by the individual; therefore, diagnosis relies on biomarker confirmation.^11^, ^15^ IWG recommendations (2021) stipulate that an AD diagnosis should be informed by biomarker evidence of pathological amyloid beta (Aβ) and tau.^11^ The NIA‐AA research framework also recognizes the importance of biomarkers supporting classification and differential diagnosis.^7^ The framework introduced the concept of amyloid, tau, and neurodegeneration (A,T, and N, respectively) biomarkers. Based on the biochemical Aβ pathway model,^16^ it is thought that the overproduction of Aβ and/or disrupted clearance of Aβ within a cascade of pathophysiological events leads to progressive Aβ plaque deposition in the brain (termed “A” in the AT(N) NIA‐AA research framework^7^).^17^ Involvement of pathologic tau (termed as “T”^7^) is believed to be a downstream effect of amyloid aggregation^17^ observed in AD.^18^ Accumulation of reactive soluble Aβ species (namely oligomers) affects synaptic plasticity, initiates neuroinflammation, and leads to tau abnormalities and neurofibrillary tangles producing cell loss and neurodegeneration (termed as “N”^7^).^17^

With validation of novel biomarkers, there is a need to refine the AT(N) classification system. In 2018, the addition of an “X” to the AT(N) framework (termed ATX(N) framework) was proposed.^19^ This addition allows for biomarkers that are non‐specific for AD but important for AD progression, such as biomarkers of neuroinflammation, oxidative stress, apoptosis, mitochondrial dysfunction, and loss of synaptic plasticity, to account for presence and/or absence of pathologies.^20^ The 2024 update for AD diagnostic and staging criteria recognized the importance of separating the clinical syndrome from the etiology as well as broadening the criteria beyond ATX(N). Because symptoms are a manifestation of the disease itself and not requisite for diagnosis, the renewed workgroup proposes a new biomarker classification system where an abnormality in “Core 1” AD biomarkers (e.g., Aβ42, p‐tau217, p‐tau181, p‐tau231) is adequate to establish an AD diagnosis. “Core 2” AD biomarkers (e.g., biofluid and tau positron emission tomography [PET]), which are often impacted later in the course of the disease, provide staging information throughout the AD continuum.^10^ Non‐core biomarkers are those not specific to AD but that are associated with a higher risk of cognitive decline and dementia. These include neurodegeneration (presently denoted as “N”, without the parenthesis) and inflammatory/immune processes (I), which may gain clinical utility over time.^10^

Validated fluid biomarkers of AD include Aβ, total tau (t‐tau), various p‐tau isoforms, as well as multiple indicators of neuronal loss, axonal compromise, synaptic loss, and neurodegeneration.^7^ Levels and changes in AD biomarkers are measured by well‐validated techniques. For instance, amyloid PET imaging has been used widely in clinical and research settings for decades to quantitatively measure fibrillar amyloid deposition.^21^ Amyloid concentrations can also be measured in the cerebrospinal fluid (CSF), with lower CSF Aβ42 levels correlating with pathological amyloid deposition in the brain.^11^ Pathological tau can be measured by the concentrations of p‐tau in plasma or CSF, as well as through tau PET imaging.^11^ Neurodegeneration, meanwhile, can be measured via CSF neurofilament light (NfL), neuroimaging, computerized tomography (CT), and magnetic resonance imaging (MRI).^10^

## 3 CHANGES IN BIOMARKERS HAVE PREDICTIVE BENEFIT

Some biomarkers of AD can predict clinical progression. Recent advances in CSF‐ and blood‐based biomarker research have, in particular, shown improved ability to detect and stage AD, as well as to predict the progression of disease (Table 1).^22^, ^23^ For instance, plasma p‐tau181 and p‐tau217 levels in individuals with MCI‐AD predict relatively near‐term cognitive decline and progression to AD dementia.^22^ In addition, concentrations of CSF Aβ42, p‐tau/Aβ42, and total tau/Aβ42 correlate with clinical decline in individuals with MCI‐AD.^22^ The potential utility of microglial and synaptic biomarkers in AD diagnosis and prediction of disease progression has recently gained traction. Microglia, for example, have been identified as molecular drivers in AD pathogenesis, whereby changes in the concentrations of microglial markers (such as Ionized calcium‐binding adaptor molecule 1 (Iba1), Transmembrane protein 119 (TMEM119), and Purinergic receptor P2Y12 (P2RY12) have been observed in patients with AD.^24^ In addition, plasma levels of the astroglia biomarker glial fibrillary acidic protein (GFAP) track disease progression and therapeutic response to amyloid reduction.^25^, ^26^ Biomarkers of synaptic pathology (e.g., neurogranin, Growth associated protein 43 (GAP‐43), Synaptosomal‐associated protein 25 (SNAP‐25), Neuronal pentraxin‐2 (NPTX2)) are correlated with disease progression or cognitive decline in AD.^27^, ^28^, ^29^ It is now evident from pivotal trials that a substantial reduction in amyloid accumulation forecasts a delay in cognitive decline.^30^ A greater understanding of the biologic changes that precede cognitive and functional deterioration in early AD may assist clinicians in assessing the appropriateness of DMT use in individuals with early AD (MCI‐AD and mild AD dementia). Such knowledge will allow clinicians to better engage with their patients on what treatment outcomes to anticipate over time.

**TABLE 1:** AD biomarkers with the potential to serve as surrogates that predict meaningful patient benefits.

CSF‐based biomarkers	Blood‐based biomarkers	Imaging markers
Aβ42^a^	Aβ42/Aβ40 ratio	Amyloid PET
Total tau p‐tau^b^	p‐tau181 p‐tau217	tau PET
NfL	NfL	–
BACE1 TREM2 YKL‐40 Neurogranin SNAP‐25 Synaptotagmin VILIP‐1	GFAP	FDG‐PET hypometabolism MRI atrophy

*Note*: Adapted from Assunção, S.S.*, et al. Alzheimers Res Ther* **14**, 54 (2022).

Abbreviations: Aβ, amyloid beta; BACE1, β‐site amyloid precursor protein cleaving enzyme 1; FDG, fluorodeoxyglucose; MRI, magnetic resonance imaging; NfL, neurofilament light; PET, positron emission tomography; p‐tau, phosphorylated tau; SNAP‐25, synaptosome‐associated protein 25; TREM2, triggering receptor expressed on myeloid cells 2; VILIP‐1, Visinin‐like protein 1; YKL‐40, Chitinase‐3‐like protein 1.

^a^ Alone or when measured as a ratio with Aβ40, total tau, or p‐tau.

^b^ Alone or when measured as a ratio with Aβ42.

## 4 UNDERSTANDING WHAT CONSTITUTES CLINICALLY AND BIOLOGICALLY MEANINGFUL BENEFITS

The concept of clinical meaningfulness differs from that of statistical significance in that the former refers to the practical importance of a treatment effect with respect to its direct impact on the patient and/or family.^31^ Clinical trial study designs stipulate a statistically significant between‐group treatment difference as a determinant of success. However, statistical significance between groups in a well‐controlled clinical trial setting is not necessarily reflective of a clinically meaningful benefit for patients in the real world.^31^

There are two important components of meaningfulness: (1) the relevance of the domains measured within trial outcome assessments to a patient's daily life; and (2) the magnitude of treatment effect, which can be described either qualitatively (e.g., based on an external reference with already established relevance) or quantitatively (e.g., based on a combination of factors, including effect size, durability of the treatment effect, and the consistency and reliability of the treatment effect in slowing or preventing disease progression).^32^, ^33^, ^34^ Due to its importance to the concept of clinical meaningfulness, we use the term “meaningful patient benefit,” which encompasses both quantitative and qualitative aspects of a meaningful benefit with patient centricity in mind.^6^ The term is broader than “clinical meaningfulness” and allows inclusion of the prediction of an increased magnitude of treatment differences over time and of therapeutic effects on biomarkers.

The urgent need for therapies that treat the underlying pathology, pathophysiological mechanisms, and biological targets that change the trajectory of AD has spurred intense, decades‐long attempts to develop DMTs for AD. Recent trials, including large‐scale, phase 3 studies, have recruited patients in the early symptomatic stages of AD, such as those with MCI‐AD.^6^, ^35^ To these patients and their care partners, the most desired outcomes (i.e., what matters most) of therapies were improvement or restoration of memory and stopping or slowing AD progression.^34^, ^36^ It is important, therefore, to assess how traditionally measured treatment effects relate to these desired outcomes.

There are limitations to using traditional clinical outcome assessments in clinical trials of early AD. For example, floor and/or ceiling effects can occur in very mildly or rapidly progressing patients. It is important to note that the mean treatment exposure in phase 3 trials of AD was 105 weeks,^6^, ^37^ which may be insufficient to extrapolate the long‐term benefit of DMTs. Moreover, cognitive or functional changes measured within the early stages of the AD continuum can be subtle.^38^, ^39^ Thus, defining what constitutes a meaningful benefit remains challenging. As a result, an integrative and comprehensive multidimensional approach is needed to define meaningful benefit in early AD. A number of solutions can be utilized to reduce these limitations and implement a structured methodology, including: (1) multidimensional clinical outcome assessments (COAs) comprising not only outcomes for cognition and function, but also neuropsychiatric symptoms (NPSs), patient‐ and care partner–reported outcomes, quality‐of‐life questionnaires, and health and economic outcomes; (2) complementary analyses (e.g., time‐to‐event [TTE] or number‐needed‐to‐treat [NNT] analyses) to contextualize the results from COAs; and (3) assessments of cumulative and predictive benefits, where early changes in cognitive, functional, or biomarker assessments predict longer‐term clinical benefit.^6^

Biomarker evidence of disease slowing is of high importance as it may suggest enduring benefit, even after stopping the therapy. Dynamic biomarker changes can serve as markers to accompany relevant clinical outcomes and buttress tangible benefits in response to a DMT. However, CSF‐based, blood‐based, and imaging biomarkers are not yet validated or qualified for use in this way, and more research is needed to incorporate them into this framework.^6^ Given the rapid expansion of AD biomarker research, well‐validated surrogate biomarkers that can reasonably predict a meaningful benefit are likely to emerge once they are fully validated in careful studies. Several AD biomarkers have been included in long‐term trials embedded in large collaborations (e.g., Alzheimer's Disease Neuroimaging Initiative [ADNI])^40^ or collected in industry‐sponsored clinical trials, and these have the potential to become well‐validated surrogate measures. Such candidates include CSF, plasma, and imaging biomarkers of Aβ protein, tau, and neurodegeneration. For example, several studies have shown that changes in amyloid deposition measured by CSF‐ and blood‐based biomarkers can predict disease progression and rate of clinical decline. Slowed disease progression is reported consistently as meaningful by individuals with AD and their care partners. Therefore, it is possible that, in the near future, patient‐centric meaningful benefits can be measured by and based on biomarker changes.^6^

## 5 ASSESSING BIOLOGICAL AND CLINICAL TREATMENT EFFECTS OVER TIME

Cumulative benefits, both biological and clinical, are important components of evaluating the clinical impact of DMTs in the earliest stages of AD. Cumulative benefits account for the amassing effects of treatment in the long term that are presumed to persist after treatment cessation.^3^, ^6^ In contrast to previously approved AD treatments that may have demonstrated improved cognition or reduction of symptoms associated with disease, DMTs elicit a persisting response (delta) and impede clinical progression of AD to later stages.^3^

**FIGURE 1:**
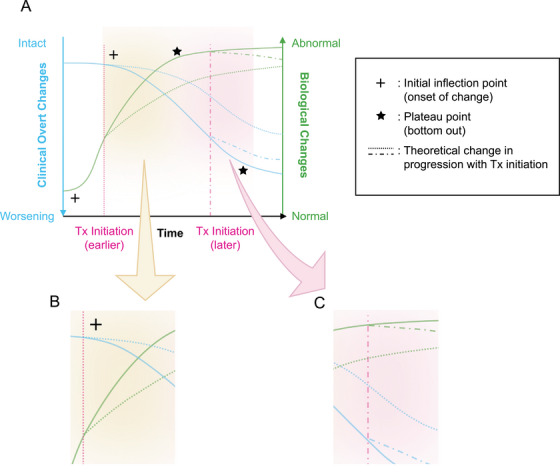
Theoretical biological and clinical changes associated with the initiation of biomarker‐guided therapy. The x‐axis indicates time; this general period/duration shows progression of clinical and biological changes and intentionally does not correlate with any disease stage. Disease stage benchmarks are highly individualized, and more data are required to better reflect clinical realities. The two y‐axes portray biological changes **(A**, right; in green) and clinical changes (left; in blue). Evidence suggests that biological changes occur earlier in the disease course than symptomatic clinical changes. This is shown with plus signs, where the biological inflection point comes before the clinical inflection point. Another consideration is the plateau (saturation) period, where biological changes also precede clinical changes: shown with stars. The dotted lines at two distinct time points indicate the theoretical changes in disease progression with continuous biomarker‐guided treatment intervention. The earlier treatment would theoretically result in a larger treatment–no treatment difference over time and considerably larger benefit. Conversely, delaying treatment initiation is expected to result in smaller treatment–no treatment differences over time. Overall, this graph takes into account the non‐linear progression of disease along with the time‐dependent deviations and cumulative changes in underlying pathophysiology and complexity. **(B)** Shows a magnified portion of A where earlier treatment would theoretically result in a larger treatment–no treatment difference over time and considerably larger benefit. **(C)** Conversely shows that delaying treatment initiation is expected to result in smaller treatment–no treatment differences over time.

DMTs show disease modification in four ways (Figure 1). First, they increase the relative difference in outcome measurements between treatment and placebo throughout the course of the trial. Second, they extend the duration in early disease stages and reduce rates of stage transition, which can lead to a lower likelihood of progression to the next global Clinical Dementia Rating (CDR) stage. Third, they demonstrate an increased amount of time preserved between treatment and placebo in TTE analyses. Finally, they slow progression of clinical decline.^3^ This disease‐modifying effect can be visualized by a change in the trajectory of decline and an increasing drug–placebo difference over time (Figure 1A).^3^, ^41^, ^42^, ^43^, ^44^ Given that cumulative benefits of DMTs are anticipated to be time dependent (e.g., an increase in benefit as the treatment duration increases),^44^ evaluating the long‐term cumulative impact of emerging AD DMTs is crucial to characterizing their benefit. If treatment effects of DMTs are likely to grow over time, a small early measurable benefit may make a larger difference for an individual over the long term (Figure 1B,C).^45^ Once this trajectory is defined, the assessment of biomarker changes may offer a means of predicting a DMT's longer‐term meaningful benefit.^6^ Further data will be needed to understand the cumulative effects of multiple treatments (e.g., AD DMTs and other agents affecting AD symptomatology).

For biomarker‐guided, targeted therapies in AD, target engagement measured by a single methodology is only one of the biological effects. Impact on other pathological pathways—both upstream and downstream—may have more impact on the disease course than the changes to the primary target itself. For example, removal of amyloid plaques as seen by PET following anti‐amyloid antibodies is only one of the biological consequences; on its own, this may not capture other important changes such as upstream alteration in soluble amyloid species or downstream changes in pathological tau and neurodegeneration.^46^ In addition to the proposed benefits of amyloid plaque removal with treatment, it is important to note the impact of downstream biomarker effects, that is, the downstream effects on plasma p‐tau, GFAP, tau PET (and clinical outcomes) that indicate a meaningful slowing of progression. It is plausible that predictive benefits may also be identified through the utility of biomarkers associated with underlying pathophysiological mechanisms of disease (i.e., Aβ42, t‐tau, p‐tau) or through clinical features (i.e., reduced cognitive or functional decline) that are measurable and quantifiable.^47^, ^48^ The true relevance and importance of such biomarkers and their clinical effects remain to be determined, and require further real‐world studies to confirm these hypotheses. Understanding the correlation between cumulative and predictive benefits may allow persons with early AD to better understand the expected biological and clinical outcomes of treatment (e.g., cognitive, behavioral, activities of daily living [ADLs], quality of life [QoL], dependency, etc.).^34^

Several analyses can help explore the meaningfulness of a therapeutic response. One such analysis is the TTE approach, which measures the length of time until an event, such as an individual's transition from one AD stage to another.^6^ Delay of disease progression is desired by patients as part of “what matters most,” but with caveats requiring large sample sizes and inter‐individual variation of TTE events.^33^ In addition to TTE approaches, other methods such as time component tests can translate the clinical scale point change into “time saved” longitudinally.^49^ In early disease stages with more subtle changes, it may be difficult to identify delayed milestones not captured through clinical rating scales.^33^ Furthermore, TTE analyses are usefully paired with real‐word evidence studies. In addition to time‐to‐progression analyses, other important milestones exist that may not depend on transition across AD stages (e.g., loss of ability to drive, read, go out unaccompanied, and remain home alone). NNT analyses can be applied to these milestones as they relate to absolute risk reduction and thus may best reflect whether a treatment and delay of a milestone was successful (i.e., a high NNT would indicate a less effective treatment).^6^ Theoretically, a DMT for AD would delay conversion from an earlier AD stage to the next and would prolong the time to key milestones (such as loss of the ability to drive).^50^ It is now evident from pivotal trials that a substantial reduction in amyloid accumulation forecasts a delay in cognitive decline.^50^

## 6 KEY TREATMENT OUTCOMES OF INTEREST TO PATIENTS

Individuals with early‐stage AD generally exhibit mild cognitive symptoms that progressively worsen and ultimately lead to increasing functional impairment and loss of independence. NPSs also intensify as the disease progresses. The increasing load of symptoms in turn translates to an increased burden for care partners as individuals progress through more advanced stages of the disease. As this occurs, individuals lose their autonomy and become more dependent on care partners. As such, to assess meaningful benefits along the AD continuum, multidimensional COAs are required.

Historically, clinical trial endpoints were designed to measure cognitive and functional changes in patients with mild to severe AD dementia. These endpoints are often not sensitive to the subtle changes that occur in the preclinical or MCI‐AD stages.^6^ For example, on the CDR global severity scale score (ranging from 0 [denoting normal cognition and function] to 3 [severe dementia]), changes between 2.0 and 3.0 (moderate to severe dementia stages) are more easily distinguishable compared with changes between cognitively unimpaired (0) to mild cognitive impairment (0.5), or from mild cognitive impairment (0.5) to mild dementia stages (1.0).^51^ By the nature of DMTs, treatments that provide subtle benefit to persons with preclinical AD and MCI‐AD may slow functional impairment and loss of independence in later stages of the disease. Hence, it is important to have sensitive and well‐validated COAs that can capture meaningful patient benefit in the early stages of AD.^38^

The FDA has provided guidance on the development, validation, and application of novel COAs to assess clinical changes in preclinical or early AD.^8^ One such COA is the Preclinical Alzheimer Cognitive Composite (PACC)^6^ and the expanded PACC5 with category fluency,^52^ which measure cognitive changes in preclinical AD in both Aβ+ and Aβ– individuals. The use of PACC5 may detect decline even in the preclinical population, indicating that semantic memory impairment is observable at the earliest, most subtle stages of disease.^52^ Composite scores such as the Alzheimer's Disease Composite Score (ADCOMS) and composite scale such as the Integrated Alzheimer's Disease Rating Scale (iADRS) can assess cognitive and functional changes that occur in early AD, and have been used in some phase 2 and 3 trials.^53^, ^54^ Another scale known as the Cognitive‐Functional Composite could improve disease progression monitoring in both trial and practice settings.^55^ To date, the majority of these scores/scales have played a role only in clinical trial settings and are not commonly used in routine clinical practice.

In addition to cognitive and functional outcomes, NPSs, patient‐ and care partner–reported outcomes, QoL, and health and economic outcomes are useful for the complete assessment of meaningful patient benefit.^6^ NPSs such as depression, anxiety, apathy, irritability, agitation, and sleep disorders are common in AD.^56^ For patients and care partners experiencing the burdensome nature of these symptoms, preventing the emergence or reducing the severity of NPSs over the course of AD may be a key measure of meaningful benefit.

Patient‐reported outcomes (PROs) are particularly important in the preclinical and early AD stages, as this is when insight is preserved and changes may be too subtle to be reliably observed by care partners or clinicians.^6^ One PRO, the Cognitive Function Index (CFI), has been shown to be sensitive to amyloid accumulation even at the preclinical stages of AD.^57^ Patient‐ and care partner–reported outcomes related to cognitive decline, compromised independence, reduced QoL, and impaired physical health have been identified by both patients and their care partners as important losses associated with progression of AD.^34^ The Patient‐Reported Outcome Consortium's Cognition Working Group developed a novel PRO measure in patients with MCI‐AD, which evaluated two functional domains—complex ADLs (e.g., financial planning, programming the television, and meal preparation) and interpersonal functioning (e.g., conversational skills and comprehension of written material)—that may deteriorate in later stages of AD.^58^ Given that the rate of functional decline accelerates along the AD continuum, tools to track incremental changes in function can be significant indicators of progression. Furthermore, functional questionnaires like the Amsterdam Instrumental Activities of Daily Living Questionnaire (A‐IADL‐Q) are sensitive to early‐stage changes and can capture the effects of early AD‐related cognitive decline on daily function.^38^, ^42^ It is important to define what is considered a meaningful decline on an IADL in early AD,^42^ given that not all IADLs are of equal importance to any given patient.

Apart from NPSs and PROs, health and economic outcomes represent another means of assessing meaningful benefit. The health and economic impact associated with AD is substantial, and it often begins even in the early stages of the disease. For example, patients may withdraw from employment due to their disease, and care partners may be required to exit the workforce due to the demands of caregiving. Various stakeholders may value different health and economic outcomes, but two major outcomes of interest are the reduction in resource utilization and retained patient autonomy, which translate into reduced care partner burden and delayed institutionalization, respectively.^6^

When considering the potential benefit of a treatment, both patients and physicians also need to consider the potential risks and when risk events may occur. Such time‐dependent perception of risk versus benefit could influence treatment choice in AD, as risk events may be immediate, whereas benefits may be observable only in the future. Because of the progressive nature of disease, and the benefits of AD DMTs being cumulative over time, the patient's perception of the immediate risks of a given treatment may discount the future long‐term benefit and thus treatment decision. Conversely, the opposite pattern has been documented for the multiple sclerosis (MS) treatment natalizumab, which can confer risk of developing progressive multifocal leukoencephalopathy (PML) in the future. One study showed that patients and physicians place greater premium on the proximal benefit of natalizumab treatment—and the benefit to mitigate MS progression—over the future risk of PML, and that patients accept the risk to greater degrees than physicians do.^59^ Such future discounting (or incentivizing) treatment choice based on risk–benefit assessment will require future studies for AD DMTs as the real world use of interventional therapeutics gain traction. Another factor in potential risk–benefit decision‐making may be the actual versus perceived burdens that are associated with treatment, such as the amount of time spent in clinics (e.g., in chemotherapy or in ambulatory care^60^) or indirect and/or societal costs estimated with slowing AD progression.^61^ Future studies are needed to empirically assess the impact of AD DMTs on the aforementioned PROs, NPSs, and burden assessment, and to account for potential risk–benefit bias in patients, as to whether the decision to initiate treatment will be affected accordingly.

The ability to detect and diagnose disease earlier can allow for reliable prediction of long‐term outcomes of treatment effects. Such tangible information is important not only to patients, but also their care partners. The role of the care partner changes along the AD continuum: in the early stages of AD, the care partner provides emotional support and assistance in future planning as the affected individuals remains relatively independent; as the disease progresses, the care partner provides more hands‐on assistance.^5^ These later disease states are associated with reduced QoL for the care partner.^62^ As such, assessing the QoL of patients as well as their care partners at each stage is crucial to providing clinicians with a more holistic understanding of the impact of AD and of potential therapeutic benefit.^5^ To achieve this, information on natural trajectories along the AD continuum is crucial yet scarce. Some initial evidence suggests that QoL decreases over time across the AD continuum of SCD, MCI‐AD, and dementia.^63^ In addition, a cross‐sectional study found that patients with dementia have less life satisfaction than at earlier stages along the continuum, largely due to diminished access to and participation in meaningful activities.^64^ Measuring the appropriate outcomes that capture data that are meaningful to patients and their care partners can provide clinicians with the confidence that a treatment has clinically meaningful benefits.

## 7 DEFINING A MINIMAL BIOLOGICALLY AND CLINICALLY IMPORTANT DIFFERENCE (MBCID)

The term minimal clinically important difference (MCID) has been widely adopted in clinical settings.^65^ The term was first coined by Jaeschke et al. in 1989, and defined as the smallest quantitative difference reflecting the patient's perception of being beneficial while also leading to a change in clinical management.^65^, ^66^ Although MCID has been measured in various clinical settings, the values have not been well‐defined in other therapeutic areas and have limitations.^65^ Variability across stages of a disease means that the MCID may differ for different patients with the same disease.^67^ Furthermore, even though the MCID score is determined per patient and is subjective, the MCID scores for a particular group of patients are averaged, thereby diminishing the patient–patient difference.^65^ Treatment effects observed in clinical trials may not necessarily translate into beneficial effects in clinical practice due to poorly chosen “landmarks” that lack relevance to patients and/or other stakeholders; this is observed not only in AD but also in other therapeutic areas.^68^ MCID approaches are particularly difficult to apply to DMTs where treatment/no treatment differences are increasing over time, and where patients who do not meet the MCID at one time point may meet or exceed the MCID later.^69^ In these cases, patients will not notice a biological improvement induced by DMTs, although the changes may have marked effects on current or future function.

The concept of MCID is valuable for evaluating the effects at the individual patient level. However, there is currently no standardized method for estimating MCID.^70^ Although there are two commonly used approaches for estimating MCID (i.e., anchor‐based and distribution‐based approaches), there is considerable variation in the resulting MCID calculations reported from various groups.^70^ For example, MCID estimates for the Mini‐Mental State Examination (MMSE) were 3.7 in one study^71^ and 1.4 in another.^72^ Furthermore, MCID uses an ordinal scale, which lacks a fixed unit and can lead to varied change scores across the scale. This constrains the stability of the MCID definition across its range and the proper interpretation of the final results, which could give rise to false‐positive or false‐negative results.^67^ These limitations can potentially be overcome by the Rasch model, which transforms ordinal data to interval data and removes the obstacle of change score variation across the scale, because the increment along an interval scale is equal for each step.^67^ As MCID is a dynamic concept, it varies for each clinical outcome assessment and according to disease stage or severity.^67^ Furthermore, MCID can differ across treatments in the same patient group.^67^ Therefore, one needs to be mindful of using previously established MCID cutoff values in new clinical trials that involve a different patient population, outcome measure, and/or treatment. The cumulative benefit of DMTs also makes applying the MCID concept challenging, since the patient would progress from not meeting, to meeting, to exceeding an MCID value as benefit accrues.^69^

As a result of the above‐noted limitations associated with MCID and due to the advances in AD biomarkers reflecting the changes associated with biological progression of the disease, the impact of a treatment on disease course and the benefit to patients may be more fully assessed if both biological and clinical differences are captured. For cancer‐related fatigue and QoL, for example, concentrations of inflammatory cytokines that can be readily quantified are associated with PROs.^73^ Determining the correlation between cytokine changes and improvements in PROs can steer treatment decision‐making as it relates to treatment type or tolerance.^73^

**FIGURE 2:**
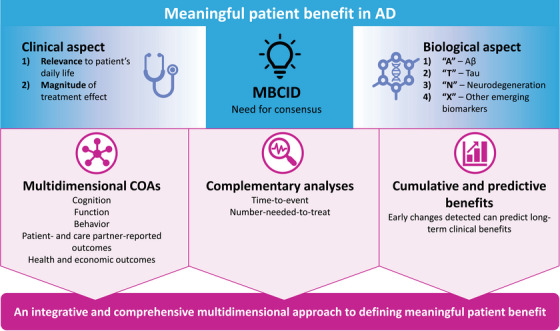
Framework for defining clinical and biological meaningfulness in Alzheimer's disease. Minimum biologically and clinically important difference (MBCID) integrates both the outcomes and observations from patients as they deem relevant, with the biological measurements that can be quantitatively measured (top row). Multidimensional clinical outcome assessments (COAs) and complementary analyses that can assess multiple domains of brain function over time (bottom row) provide a comprehensive approach to define what is a meaningful benefit of AD therapeutics.

We posit that a more complete understanding of benefit in the AD therapeutic assessment would be achieved with the concept of a minimum biologically and clinically important difference (or MBCID) (Figure 2). Like the MCID, MBCID would quantify the smallest changes in biological and clinical outcomes that are important for a particular patient, which is different from a between‐group treatment effect. Because biological differences and clinical differences are not synonymous, it is crucial that any incremental change is captured for better precision of future outcomes. An important theoretical assumption for DMTs on the longitudinal benefit for patients is that the extent of biologically and clinically important differences is a function of treatment onset and treatment duration; these must reflect non‐linear progression of disease, with time‐dependent, cumulative changes in underlying pathophysiology and complexity. Furthermore, the heterogeneous nature of AD and associated differential responses require prediction of outcomes and evaluating treatment response at an individual level.^74^ The success of treatment can be measured by the proportion of patients who reach the MBCID as opposed to the average change in a group of patients.^75^

A preliminary example of an MBCID is a recent study demonstrating that p‐tau217 predicted the emergence of AD dementia accurately but that the accuracy was improved by adding measures of cognition as well as the apolipoprotein E (*APOE*) genotype.^76^ This is a biomarker and cognitive outcome composite that has prognostic value, and, if confirmed, functions as an MBCID. With additional real‐world evidence, a variety of MBCIDs may be identified utilizing different biomarker and clinical measure combinations.

Biomarkers are more sensitive than clinical measures and can change with little or no corresponding clinical impact.^6^ Biomarkers can complement clinical measures by quantifying AD‐related pathological changes, even at early stages of disease when these measures might have limited sensitivity.^5^ The five major biomarkers and supporting diagnostic modalities of AD (namely CSF Aβ42, amyloid PET, CSF p‐tau, structural MRI, and fluorodeoxyglucose–positron emission tomography [FDG‐PET]) denoted in the staging progression model of AD biomarkers reflect pathophysiological processes and temporal associations between the presence of underlying AD biomarker changes and the onset of clinical symptoms.^77^ Biomarker testing may be particularly useful along the center portion of the sigmoidal curves, where relative changes can be quantified and compared over time. To date, interventional clinical trials have shown that the trajectory of biomarkers can shift bidirectionally in time, whereas curves denoting clinical function may flatten or decrease over time. Thus this dynamic characteristic would be meaningful for target engagement to predict the trajectory of clinical progression when used in conjunction with clinical features.^5^ Because biological changes are not perceived by the patient, the treating clinician may use the biological landmarks as a surrogate for observable changes and as a potential predictor of future benefits such as delay to onset or worsening of deficits.

Given that measuring biological changes may mitigate the MCID limitations by quantitatively and objectively measuring improvements and reducing patient–patient variability, the use of an MBCID may more accurately reflect fundamental change for the patient. Progressor analyses may be used to help define meaningful progression on COAs by demonstrating the proportion of patients who progress with treatment compared with placebo.^6^ In the context of treatment response, it remains to be seen whether responder analyses can help to demonstrate the proportion of patients who respond to treatment (i.e., achieve or exceed the within‐patient meaningful change threshold).^6^

## 8 NEED FOR CONSENSUS IN DEFINING BIOLOGICAL AND CLINICAL MEANINGFULNESS AND MBCID

Assessing trial outcomes via a comprehensive evaluation of cognitive, functional, and behavioral changes—along with PROs, resource utilization endpoints, and disease progression biomarkers—will allow better interpretation of an AD treatment's true benefit. As more trials include biomarkers as well as comprehensive clinical outcome measures, more data will be collected to improve understanding of the trajectory of biomarker change in relation to treatment response and clinically meaningful benefits. The EU/US Task Force considers biomarker evidence, especially when combined with a single clinical trial outcome, as supportive of the meaningful benefits of a treatment.^78^ According to the Task Force, amyloid and tau biomarkers were regarded as providing mild to moderate support for disease modification; moreover, combinations of tau and neurodegeneration biomarkers were regarded as providing moderate to marked support for disease modification, and combinations of amyloid, tau, and neurodegeneration biomarkers were regarded as providing marked support for disease modification.^78^

It is essential to consider the treated individual's perception of what constitutes a sufficient impact on their current or future health status or what outcome domains they feel best signify a treatment benefit.^67^ Informant‐rated measures of everyday function are increasingly being included as endpoints in clinical trials due to their potential prognostic value. One example is the Everyday Cognition (ECog) Performance Status Scale, which was developed to detect mild functional changes that are related to cognitive impairment and which may predate loss of independence or decline in major ADLs.^79^, ^80^ The ECog scale is sensitive to longitudinal changes along the AD continuum and has been associated with AD biomarkers and objective measures of cognition.^81^, ^82^ Clinically meaningful change estimates considered by clinicians to be appropriate for the detection of changes that occur in the early stages of AD have been reported.

MCID values have been calculated for most COAs used in clinical trials. The MCID cutoff values for ADCOMS change score were 0.05 and 0.10 in patients with MCI due to AD and mild AD, respectively;^83^ for the iADRS, these values were 5 and 9 points, respectively.^54^ MCID cutoff values have also been reported for three assessment tools that are commonly used in clinical trials (i.e., the MMSE, CDR‐SB (Sum of Boxes), and Functional Activities Questionnaire), but these values reflect the range of clinically meaningful change estimates across the whole AD continuum (i.e., not just MCI‐AD and mild AD, but also moderate‐to‐severe AD).^70^ Notably, the National Alzheimer's Coordinating Center (NACC) database used in the study may not be representative of the broader AD population due to differences in patient characteristics and enrollment procedures. In addition, the diagnostic criteria for MCI‐AD in this study did not reflect that of current clinical practice and a substantial population was not biomarker‐verified as AD.

## 9 UTILIZING TREATMENT BENEFITS DETECTED IN EARLY STAGES OF DISEASE TO MEASURE OUTCOMES ACROSS THE AD CONTINUUM

Core clinical features commonly assessed in AD trials are cognition, function, and behavior, which are differentially affected across the AD continuum.^5^ Several clinical measures have been used as trial outcomes to assess these clinical features. However, many existing clinical assessment tools require expert administrative experience and have limited sensitivity to subtle changes in the preclinical or early stages of AD, where patients tend to exhibit slow and variable progression.^5^ These can be exacerbated by high variability in baseline functioning, cognitive ability, and educational and cultural backgrounds of patients, as well as floor and/or ceiling effects of many of the clinical assessment tools.^5^ Similarly, many common scales used in clinical practice lack sensitivity to detect change in patients with biomarker confirmation of AD but few symptoms.^45^

Although a variety of tools are available for use in clinical trials for early AD, not all of them are practical for use in real‐world clinical practice (and vice versa). For example, the Montreal Cognitive Assessment (MoCA) is commonly used in routine clinical practice to assess cognitive changes in early AD, but it is not widely used in clinical trials as an outcome assessment.^5^ On the other hand, the Alzheimer's Disease Assessment Scale–Cognitive Subscale 13 items (ADAS‐Cog‐13), a modified version of the ADAS‐Cog‐11 that includes a delayed word recall task and a number cancellation task to increase the sensitivity of the scale to mild AD, is widely used in clinical trials but not in clinical practice.^5^

For the detection of functional changes in early AD, the European Medicines Agency (EMA) recommends the use of Alzheimer's Disease Cooperative Study‐Activities of Daily Living Scale‐Mild Cognitive Impairment (ADCS‐ADL‐MCI) and A‐IADL‐Q over the assessment of basic activities of daily living (BADL). ADCS‐ADL‐MCI and A‐IADL‐Q are sensitive to longitudinal changes of IADLs along the AD continuum, whereas BADLs are affected in the later stages of AD.^5^, ^38^, ^42^, ^84^ For the detection of behavioral changes in early AD, the Neuropsychiatry Inventory (NPI)^85^ and/or NPI‐Q, and the Mild Cognitive Behavioral Impairment Checklist^86^ can help guide the assessment of neuropsychiatric symptoms in the early stages of AD. Composite scores and scales of cognition and function that have shown sensitivity to changes in early AD include the CDR‐SB, ADCOMS, and iADRS (Table 2).^5^

**TABLE 2:** Clinical outcome assessments that can assess clinical changes in preclinical AD or MCI‐AD.

Domain	Assessment	Preclinical AD	MCI‐AD
**Cognition**	PACC	✓	
	APCC	✓	
	ADAS‐Cog‐13		✓
	NTB		✓
	MMSE		✓
	MoCA		✓
	RBANS		✓
	CANTAB		✓
	Cogstate		✓
**Function**	UPSA	✓	
	VRFCT	✓	
	ECog	✓	
	CFI	✓	
	ADCS‐ADL‐PI	✓	
	ADCS‐ADL‐MCI		✓
	A‐IADL‐Q		✓
	FAQ		✓
**Behavior**	NPI		✓
	MBI‐C		✓
	BEHAVE‐AD		✓
**Global/composite**	ADCOMS	✓	✓
	CDR‐SB		✓
	iADRS		✓
	CFC		✓
	CIBIC Plus		✓
**Health and economic outcomes**	RUD Questionnaire		✓

Abbreviations: ADAS‐Cog‐13, Alzheimer's Disease Assessment Scale Cognitive Subscale 13 items; ADCOMS, Alzheimer's Disease Composite Score; ADCS‐ADL‐MCI, Alzheimer's Disease Cooperative Study‐Activities of Daily Living Scale‐Mild Cognitive Impairment; ADCS‐ADL‐PI, Alzheimer's Disease Cooperative Study‐Activities of Daily Living Scale‐Prevention Instrument; A‐IADL‐Q, Amsterdam IADL Questionnaire; APCC, Alzheimer's Prevention Initiative Composite Cognitive Test; BEHAVE‐AD, Behavioral Pathology in Alzheimer's Disease; CDR‐SB, Clinical Dementia Rating‐Sum of Boxes; CFC, Cognitive‐Functional Composite; CFI, Cognitive Function Index; CIBIC Plus, Clinician's Interview Based Impression of Change with caregiver input; ECog, Everyday Cognition scale; FAQ, Functional Activities Questionnaire; MBI‐C, Mild Cognitive Behavioral Impairment Checklist; iADRS, Integrated Alzheimer's Disease Rating Scale; MMSE, Mini‐Mental State Examination; MoCA; Montreal Cognitive Assessment; NPI, Neuropsychiatric Inventory; NTB, Neuropsychological Test Battery; PACC, Preclinical Alzheimer Cognitive Composite; RBANS, Repeatable Battery for the Assessment of Neuropsychological Status; UPSA, University of California San Diego Performance‐Based Skills Assessment; RUD, Resource Utilization in Dementia; VRFCT, Virtual Reality Functional Capacity assessment Tool.

The use of DMTs is expected to slow disease progression, and therefore these assessment scores should remain stable or decline modestly for substantial periods. Relative stability of cognition and function may be an indicator of successful treatment and may be used as a way to assess and communicate meaningfulness of treatment effects in early AD stages.^6^

## 10 FUTURE OUTLOOK

Cumulative evidence suggests that a crucial step in the onset of AD is dysregulation, compensation, and decompensation in Aβ homeostasis. These produce a cascade of neuronal and glial molecular events that ultimately lead to complex systems failure with cognitive deficits (MCI‐AD) and ultimately the late‐stage AD dementia syndrome.^16^ A clearer picture of disease progression and biologically meaningful treatment‐related change, conceptually capturing target engagement, mechanism of action, impact on mechanism of disease, and surrogate function, may complement clinical outcomes in future clinical trials by serving as additional evidence of biological disease modification.

Future precision medicine in AD and in neurology more broadly may also be facilitated by differentiated multimodal and multidomain outcome development across the AD continuum. This can be achieved by taking into account meaningful subsets and groups (e.g., biological staging, molecular subtyping, genetics, ethnicities, sex, and other classifiers) as well as research into subgroups.^4^ Additional long‐term follow‐up of real‐world evidence‐generation studies is required to establish, develop, validate, and standardize biologically and clinically meaningful outcomes.^5^ The ROADMAP (Real‐world Outcomes across the AD continuum for better care: Multi‐modal Data Access Platform) project aims to evaluate the feasibility of using real‐world evidence to identify key disease and patient outcomes to enable stakeholders to make informed funding and treatment decisions. The project also aims to provide advice on data integration methods and standards, and to develop conceptual cost‐effectiveness and disease models to assess whether early treatment provides long‐term benefit.^38^, ^87^ There is a need for registries (e.g., Alzheimer’s Network for Treatment and Diagnostics (ALZ‐NET), A personalized medicine approach for Alzheimer’s disease (ABOARD)) to act as a means of capturing more uniform data across therapies in the real world.

Digital biomarkers, such as data related to executive function, movement, behavior, and sleep patterns collected using mobile/wearable devices, hold promise as potential endpoints for use future in clinical trials and clinical practice.^88^, ^89^ Digital biomarkers can enable the large‐scale and non‐invasive collection of passive and objective data on potentially clinically meaningful changes in the day‐to‐day living of individuals with AD in a manner that is minimally burdensome to the person, care partner, and health care provider.^88^, ^89^ Nonetheless, it is essential to comprehensively validate emerging digital biomarkers prior to use in clinical trials or clinical practice.^5^ The emergence of powerful computing tools in artificial intelligence (AI) has the potential to expose hidden insights into treatment responses by revealing patterns in large data sets.^4^ These tools leverage large and complex data sets from digital tools, and potentially devise prediction modeling to provide patients and their care partners with accurate prognostic information. Such AI and digital application may support care‐related decision‐making and health care planning, as demonstrated in two recent studies that developed models to predict QoL trajectories, institutionalization, and mortality for patients with SCD and MCI using clinical characteristics and AD biomarkers.^63^, ^90^ Prediction modeling may enable monitoring of outcomes that patients find important. As such, it holds promise in facilitating a shift toward value‐based and precision medicine health care. Collectively, the direction of the field in placing patient perspective of treatment benefit at the center of clinical trial design is an essential first step to ultimately define meaningful biological and clinical benefits in AD. These are necessary developments that subsequently promote more personalized and ultimately precision medicine in the clinical care pathway for patients with AD.^4^

## CONFLICT OF INTEREST STATEMENT

Harald Hampel (H.H.) is an employee of Eisai and serves as reviewing editor for the journal *Alzheimer's and Dementia*. H.H. is inventor of 11 patents and has received no royalties for: In Vitro Multiparameter Determination Method for the Diagnosis and Early Diagnosis of Neurodegenerative Disorders patent no. 8916388; In Vitro Procedure for Diagnosis and Early Diagnosis of Neurodegenerative Diseases patent no. 8298784; Neurodegenerative Markers for Psychiatric Conditions publication no. 20120196300; In Vitro Multiparameter Determination Method for the Diagnosis and Early Diagnosis of Neurodegenerative Disorders publication no. 20100062463; In Vitro Method for the Diagnosis and Early Diagnosis of Neurodegenerative Disorders publication no. 20100035286; In Vitro Procedure for Diagnosis and Early Diagnosis of Neurodegenerative Diseases publication no. 20090263822; In Vitro Method for the Diagnosis of Neurodegenerative Diseases patent no. 7547553; CSF Diagnostic in Vitro Method for Diagnosis of Dementias and Neuroinflammatory Diseases publication no. 20080206797; In Vitro Method for the Diagnosis of Neurodegenerative Diseases publication no. 20080199966; Neurodegenerative Markers for Psychiatric Conditions publication no. 20080131921; and Method for Diagnosis of Dementias and Neuroinflammatory Diseases Based on Increased Procalcitonin in Cerebrospinal Fluid: US patent no. 10921330. Sharon Cohen (S.C.) has performed consulting activities (no personal fees) with the following companies: Alnylam, Biogen, Biohaven, Cassava, Cognivue, Cogstate, Eisai, Eli Lilly, INmune Bio, Lundbeck, Novo Nordisk, ProMIS Neurosciences, RetiSpec, Roche, and SciNeuro; and research grants (paid to institution only): AbbVie, AgeneBio, Alector, Alnylam, Alzheon, Anavex, Biogen, Cassava, Eli Lilly, Eisai, Janssen, Novo Nordisk, RetiSpec, Roche, and UCB Biopharma. Jeffrey Cummings (J.C.) has provided consultation to Acadia, Actinogen, Acumen, AlphaCognition, ALZpath, Aprinoia, AriBio, Artery, Biogen, Biohaven, BioVie, BioXcel, Bristol‐Myers Squib, Cassava, Cerecin, Diadem, Eisai, GAP Foundation, GemVax, Janssen, Jocasta, Karuna, Lighthouse, Lilly, Lundbeck, LSP/eqt, Merck, NervGen, New Amsterdam, Novo Nordisk, Oligomerix, Optoceutics, Ono, Otsuka, Oxford Brain Diagnostics, Prothena, ReMYND, Roche, Sage Therapeutics, Signant Health, Simcere, sinaptica, Suven, TrueBinding, Vaxxinity, and Wren pharmaceutical, assessment, and investment companies. J.C. is supported by NIGMS grant P20GM109025; NINDS grant U01NS093334; NIA grant R01AG053798; NIA grant P30AG072959; NIA grant R35AG71476; NIA R25 AG083721‐01; Alzheimer's Disease Drug Discovery Foundation (ADDF); Ted and Maria Quirk Endowment; and Joy Chambers‐Grundy Endowment. J.C. owns the copyright of the Neuropsychiatric Inventory. J.C. has stocks/options in Artery, Vaxxinity, Behrens, Alzheon, MedAvante‐Prophase, and Acumen. The research programs of Wiesje M. van der Flier (W.F.) have been funded by ZonMW, NWO, EU‐JPND, Alzheimer Nederland, Hersenstichting CardioVascular Onderzoek Nederland, Health∼Holland, Topsector Life Sciences & Health, stichting Dioraphte, Gieskes‐Strijbis fonds, stichting Equilibrio, Edwin Bouw fonds, Pasman stichting, stichting Alzheimer & Neuropsychiatrie Foundation, Philips, Biogen MA Inc, Novartis‐NL, Life‐MI, AVID, Roche BV, Fujifilm, Eisai, and Combinostics. W.F. holds the Pasman chair and is recipient of ABOARD, a public–private partnership receiving funding from ZonMW (#73305095007) and Health∼Holland, Topsector Life Sciences & Health (PPP‐allowance; #LSHM20106). All funding is paid to her institution. W.F. has been an invited speaker at Biogen MA Inc, Danone, Eisai, WebMD Neurology (Medscape), NovoNordisk, Springer Healthcare, NovoNordisk, and European Brain Council; all funding is paid to her institution. W.F. is a consultant to Oxford Health Policy Forum CIC, Roche, Biogen MA Inc, and Eisai; all funding is paid to her institution. W.F. participated in advisory boards of Biogen MA Inc, Roche, and Eli Lilly; all funding is paid to her institution. W.F. is member of the steering committee of PAVE and Think Brain Health. W.F. was associate editor of *Alzheimer, Research & Therapy* in 2020/2021. W.F. is associate editor at *Brain*. Paul Aisen (P.A.) has research grants from National Institutes of Health (NIH), the Alzheimer's Association (AA), Janssen, Lilly, and Eisai, and consults with Merck, Bristol Myers Squibb, Switch Therapeutics, NewAmsterdam Pharma, Roche, Genentech, Abbvie, Biogen, ImmunoBrain Checkpoint, and Arrowhead. Aya Elhage (A.E.), Min Cho (M.C.), and Joanne Bell (J.B.) are employees of Eisai Inc. Author disclosures are available in the supporting information.

## Supporting information



Supporting information
